# Longitudinal data methods for evaluating genome-by-epigenome interactions in families

**DOI:** 10.1186/s12863-018-0642-7

**Published:** 2018-09-17

**Authors:** Justin C. Strickland, I-Chen Chen, Chanung Wang, David W. Fardo

**Affiliations:** 1 Department of Psychology, College of Arts and Sciences, University of Kentucky, 171 Funkhouser Drive, Lexington, KY 40506 USA; 2 Department of Biostatistics, College of Public Health, University of Kentucky, 725 Rose St, Lexington, KY 40536 USA; 3 Department of Biology, College of Arts and Sciences, University of Kentucky, 334 T.H. Morgan Building, Lexington, KY 40506 USA

**Keywords:** Change, Epigenetics, Family, Longitudinal, GEE, Mixed model, Power, QIF

## Abstract

**Background:**

Longitudinal measurement is commonly employed in health research and provides numerous benefits for understanding disease and trait progression over time. More broadly, it allows for proper treatment of correlated responses within clusters. We evaluated 3 methods for analyzing genome-by-epigenome interactions with longitudinal outcomes from family data.

**Results:**

Linear mixed-effect models, generalized estimating equations, and quadratic inference functions were used to test a pharmacoepigenetic effect in 200 simulated posttreatment replicates. Adjustment for baseline outcome provided greater power and more accurate control of Type I error rates than computation of a pre-to-post change score.

**Conclusions:**

Comparison of all modeling approaches indicated a need for bias correction in marginal models and similar power for each method, with quadratic inference functions providing a minor decrement in power compared to generalized estimating equations and linear mixed-effects models.

## Background

Longitudinal measurement provides numerous benefits for evaluating the association between genetic variation and human health. For example, the inclusion of multiple time points allows for the prospective study of time-varying covariates and an improved understanding of disease and trait progression over time. However, the promise of applying longitudinal data to genetic-based problems is tempered by the analytic challenges inherent to the approach. Particularly when incorporating related subjects, statistical methods should account for both the within-family correlation structure and the correlation among repeated measurements. The added complexity of large genetic data sets and influence of family structure makes computational limitations an additional barrier to consider. When global DNA methylation data are added to traditional candidate single-nucleotide polymorphism (SNP) genotypes these analytic and computational concerns are exacerbated.

Linear mixed effects (LME) models and generalized estimating equations (GEEs) are two broad and commonly used strategies for analyzing clustered/longitudinal data. In LME models, beyond parameterizing the correlation structure of repeated measurements, the correlations induced by familial relationships can also be explicitly modeled. This is often done using a kinship matrix to parameterize the degree of within-family relatedness. GEEs can also be used for analyzing individual-level outcomes within any given family (or cluster) assumed to be correlated [1]. When the mean structure is correctly specified, consistent regression parameter estimates are obtained, even if the working correlation structure is misspecified. However, accurately modeling the working correlation structure can be crucial in terms of estimation efficiency [2]. A method that has the potential to improve efficiency compared to GEEs is the use of quadratic inference functions (QIFs) [3]. Relative to the performance of GEEs, the QIF approach is more efficient when the working correlation structure is incorrect, whereas the 2 approaches are equally efficient once the structure is correctly specified [4, 5]. When cluster sizes vary and the number of clusters is not large, as is the case in our simulations, QIFs might produce estimates with larger variability [6].

Although longitudinal and other multivariate methods have been considered and compared when evaluating SNP genotypes in previous Genetic Analysis Workshops (GAWs) [7–9], the relative power and Type I error rates of these methods have not been extensively studied for methylation outcomes with family-based studies. Furthermore, researchers have yet to compare GEE and QIF methods for analyzing genetic data. Here, we evaluate 3 methods (LME, GEEs, and QIFs) that account for clustered measurements and test epigenetic-by-genetic interactions. We consider a conditional (baseline-adjusted) model given the nature of the data simulation process in which pretreatment values were anchored to posttreatment mean outcomes. We also consider a change score setting as a comparison method. These simplified models mean that the temporal nature of the study was not included in the design explicitly and we instead focus on a more general multivariate condition that includes repeated measurements.

## Methods

We used pharmacoepigenetic data from 200 simulated posttreatment replicates from the GAW20 data set based on the Genetics of Lipid Lowering Drugs and Diet Network (GOLDN) study. Simulation solutions were known prior to analysis. Details regarding the GOLDN study from which these data were simulated are provided elsewhere [10]. We examined interactions between 10 SNPs and the corresponding nearby cytosine-phosphate-guanine (CpG) markers. Five SNP sites were simulated to exhibit a large effect on triglyceride (TG) response [11]. Each site was simulated with varying expected heritability (h_g_^2^ = 0.025 to 0.125). The causal SNP effect was conditionally related to methylation at the nearby CpG site, with complete expression when that CpG site was fully unmethylated and complete suppression when fully methylated. The other 5 SNPs tested were noncausal and their corresponding CpG sites shared a simulated random variability with the 5 simulated causative CpG markers. This analysis included 680 individuals from 164 families with complete data at each site tested.

Separate models tested the interaction of CpG-by-SNP site (interaction test = CpG × SNP). The methylation site analyzed was posttreatment CpG located closest to the candidate SNPs. Both SNP and CpG sites were standardized prior to analysis. All analyses also included the covariates age, sex, and study center. Therefore, the full model included the CpG × SNP interaction, its constituent single-order terms, and the other covariates (age, sex, and study site). We address two different outcome settings: (a) posttreatment TGs, using pretreatment TGs as a model covariate (henceforth referred to as “Pre/Post”) and (b) a post/pretreatment TG score (henceforth referred to as “Change”). These settings mean that the temporal nature of the study was not explicitly included in the design and was evaluated as a more general multivariate rather than longitudinal method. A log transformation was applied to TG values prior to all calculations.

### LME approach

First, we used LME modeling to evaluate TG treatment response. The LME model assumed CpG, SNP, and other covariates as fixed effects and a random effect of family parameterized by the kinship correlation matrix obtained using the *make kinship* function of the “kinship2” package in R [12]. The *lmekin* function of the “coxme” package in R incorporated this kinship matrix for use in the LME model [13]. In contrast to the later GEE approaches that assume a single random effect for family, this more precisely models relationships within families.

### GEE approach

Next, GEE was used to examine TG treatment response. The observed outcome vector for the *i*th family with *n*_*i*_ members is denoted by ${\boldsymbol{TG}}_i={\left[{TG}_{i1},\dots, {TG}_{i{n}_i}\right]}^T,i=1,\dots, N$ (total number of families), which has a marginal mean given by *E*(***TG***_*i*_| ***X***_*i*_) ***= μ***_*i*_ linked to covariates though the identify function, $f\left({\mu}_{ij}\right)={\mathbf{x}}_{ij}^T\beta, j=1,\dots, {n}_i$, for **x**_*ij*_ = [1, *x*_1*ij*_, …, *x*_(*p* − 1)*ij*_]^*T*^ and *β* = [*β*_0_, *β*_1_, …, *β*_*p* − 1_]^*T*^, where *p* is the number of model covariates. The working covariance matrix for ***TG***_*i*_ is represented as ${\boldsymbol{V}}_i={\boldsymbol{A}}_i^{1/2}{\boldsymbol{R}}_i{\boldsymbol{A}}_i^{1/2}$ where ***A***_*i*_ is a diagonal matrix denoting the working marginal variances, and ***R***_*i*_ is a symmetric and positive definite working correlation matrix with 1 along the diagonal elements. Let ***D***_*i*_ = *∂****μ***_*i*_/*∂β*^*T*^ and to obtain the regression parameter estimates, $\widehat{\beta}$, using the GEE approach [1] for marginal analysis, we iteratively solve

1 $$ \sum \limits_{i=1}^N{\boldsymbol{D}}_i^T{\boldsymbol{A}}_i^{-\frac{1}{2}}{\boldsymbol{R}}_i^{-1}{\boldsymbol{A}}_i^{-\frac{1}{2}}\left({\boldsymbol{Y}}_i-{\boldsymbol{\mu}}_i\right)=0 $$

An exchangeable working correlation structure, rather than an independence structure [8], was employed. Notably, the empirical covariance might be inflated compared to its theoretical value despite both covariance matrices being asymptotically equivalent. As a result, Wald statistics are inflated and confidence intervals are overly narrow [14]. We incorporate the bias-corrected method of Mancl and DeRouen [15] to account for the bias for small sample size from $\left({\boldsymbol{TG}}_i-{\widehat{\boldsymbol{\mu}}}_i\right){\left({\boldsymbol{TG}}_i-{\widehat{\boldsymbol{\mu}}}_i\right)}^T$ or ${\widehat{\boldsymbol{e}}}_i{{\widehat{\boldsymbol{e}}}_{\boldsymbol{i}}}^T,i=1,\dots, N$, in which the estimated residuals, ${\widehat{\boldsymbol{e}}}_i$, are relatively small on average [15].

### QIF approach

The QIF approach is based on GEE [1] and the generalized method of moments (GMM) approaches [16]. It rewrites ${\boldsymbol{R}}_i^{-1}={\sum}_{r=1}^m{\alpha}_{ri}{\boldsymbol{M}}_{ri}$ in eq. (1), where ***M***_*ri*_, *r* = 1, …, *m*, *i* = 1, …, *N*, are known basis matrices and *α*_*ri*_, *r* = 1, …, *m*, *i* = 1, …, *N*, are functions of correlation parameters [3]. This approach treats GEE as a linear combination of *m* sets of unbiased estimating equations, and uses the 2-step GMM approach for extended score equations to obtain regression parameter estimates. For exchangeable covariance, ***M***_1*i*_ is an identity matrix and ***M***_2*i*_ is a matrix with 0 on the diagonal and 1 elsewhere. Regression parameter and standard error estimates are consistent even when the working correlation structure is incorrect. We note that bias-corrected methods, such as the ones discussed for the GEE approach, can also be applied to the QIF approach [15, 17]. The R code to analyze the marginal models with the GEE and QIF approaches may be found in the supporting information of previous research [18].

### Power

Each of the 5 SNP–CpG interactions from the simulation that were simulated causal for TG response were tested using a nominal 5% significance threshold. A Bonferroni correction was also calculated based on the genome-wide methylation data to increase generalizability to the typical experimental setting. This resulted in a significance threshold of 1.08 × 10^− 7^ (0.05/461281 CpG sites) [10]. Empirical power was calculated as the number of times the causal site was significant out of the total number of simulations.

### Type I error rate

To evaluate false-positive rates, we examined the 5 simulated noncausal SNP–CpG interactions. A nominal 5% significance threshold was first used. The same Bonferroni correction was then applied for genome-wide correction. The Type I error rate was computed by recording the number of times the noncausal loci were significant across all tested sites and simulations.

## Results

Table 1 contains the power and Type I error rates for each approach and outcome setting (ie, Pre/Post vs Change). At all causal sites, the Pre/Post LME model conferred greater power than the Change setting. Power was, as expected, systematically related to simulated h_g_^2^, with > 90% power observed at the chromosome 1 site (h_g_^2^ = 0.125) but only 29% power observed at the chromosome 10 site (h_g_^2^ = 0.025). Both LME settings adequately controlled for Type I error rates, with the Change setting proving a more conservative test (α = 0.038) than the Pre/Post test (α = 0.048).

**Table 1:** Power and Type I error rates for gene by methylation interactions

Model	Outcome	Nominal (*p* < 0.05)						Genome-wide (*p <* 1.08 × 10^− 7^)					
		1 (0.125)	8 (0.10)	6 (0.075)	17 (0.05)	10 (0.025)	Type I error	1 (0.125)	8 (0.10)	6 (0.075)	17 (0.05)	10 (0.025)	Type I error
LME	Pre/Post	0.930	0.790	0.690	0.545	0.290	0.048	0.016	0.020	0.000	0.000	0.000	0.000
	Change	0.839	0.715	0.595	0.510	0.210	0.038	0.011	0.005	0.000	0.000	0.000	0.000
GEE	Pre/Post	0.935	0.835	0.705	0.555	0.290	0.073	0.022	0.025	0.005	0.000	0.000	0.002
	Change	0.855	0.735	0.565	0.535	0.240	0.069	0.016	0.005	0.000	0.000	0.000	0.001
GEE–BC	Pre/Post	0.914	0.835	0.630	0.530	0.265	0.051	0.011	0.015	0.000	0.000	0.000	0.001
	Change	0.828	0.715	0.540	0.500	0.210	0.052	0.011	0.005	0.000	0.000	0.000	0.001
QIF	Pre/Post	0.941	0.845	0.685	0.580	0.410	0.120	0.054	0.055	0.015	0.000	0.005	0.002
	Change	0.860	0.735	0.575	0.580	0.345	0.111	0.048	0.015	0.000	0.000	0.000	0.002
QIF–BC	Pre/Post	0.882	0.760	0.520	0.460	0.205	0.043	0.005	0.010	0.000	0.000	0.000	0.000
	Change	0.780	0.670	0.450	0.450	0.180	0.042	0.005	0.005	0.000	0.000	0.000	0.000

*BC*, Bias-corrected methods; Change, post-pre change triglyceride score as the outcome; *GEE*, generalized estimating equations; *LME*, linear mixed effects; Pre/Post, posttreatment triglyceride as the outcome and pretreatment as a baseline covariate; *QIF*, quadratic inference functions

The proportion of 200 (or 186 in the case of the chromosome 1 site) replicates that each SNP × CpG interaction was identified as significant for each significance threshold. Presented is the chromosome location with simulated expected heritability (h_g_^2^) in parentheses

Causal chromosome sites simulated include: Chr 1 (SNP: rs9661059; CpG: cg00000363); Chr 8 (SNP: rs1012116; CpG: cg18772399); Chr 6 (SNP: rs736004; CpG: cg10480950); Chr 17 (SNP: rs4399565; CpG: cg01242676); and Chr 10 (SNP: rs10828412; CpG: cg00045910)

A similar pattern of results was observed when testing the GEE and QIF models. Again, in all instances, the Pre/Post setting provided improved power compared to the Change setting. Consistent improvements in power with increases in simulated h_g_^2^ were also observed. Both GEE and QIF at the two outcome settings adequately controlled for Type I error rates after applying the bias-correction methods. Removal of the bias-correction methods improved power, particularly for the QIF models, but also resulted in inflated Type I error rates.

Few causal sites were identified as significant after applying the genome-wide correction across all models and settings (power ≤ 5% across all cases).

Run times were estimated as 0.5, 1.25, and 0.4 s/site for the LME, GEE, and QIF models, respectively. Consequently, epigenome-wide testing (ie, 461,281 CpG sites) would take approximately 64, 160, and 52 h for the LME, GEE, and QIF models, respectively.

## Discussion

The primary purpose of the present analysis was to evaluate 3 methods (LME, GEE, and QIF) for analyzing clustered, family-based data, including genomic and epigenomic measures. Acceptable power was generally observed across the 3 methods and 2 outcome settings when the effect sizes were large (ie, simulated h_g_^2^ was high) and a nominal significance threshold was applied. Covarying baseline performance provided greater power and more accurate control of Type I error rates than computation of a pre-to-post change score in all instances. This outcome suggests that settings similar to those typically used for more complex longitudinal designs involving multiple time points will provide comparable or greater power as change score computation. Note, however, that this is also a function of the simulation structure and needs to be evaluated with other models of data generation. Comparisons between baseline adjustments and change scores for commonly employed longitudinal methods have been examined extensively in other settings [19].

After genome-wide correction, low power was observed at all sites, settings, and models tested. This outcome was not surprising given the relatively small sample analyzed, and reinforces the need for large samples and the development of more powerful methods for analyzing genome-wide genomic and epigenomic outcomes in families. Also, the limited resolution resulting from the number replications diminished our ability to appropriately test genome-wide Type I error.

Here, we evaluate for the first time QIF models for use with genetic data. Comparison of all models and settings suggested comparable power across methods, with QIF providing a slight decrement in some cases compared to GEE and LME. These findings are consistent with previous studies evaluating LME and GEE techniques for analyzing genetic data [20] and extend them to the QIF method. We also incorporated bias-correction [14, 15, 17] and found notable improvements in Type I error rates for both GEE and QIF methods. Although QIF methods gain advantage in estimation inference under moderately sized samples when employing the incorrect covariance structure, the slight decrease in power observed here may be a result of varying cluster size (ie, family size) and consequent reductions in precision [6].

Some limitations must be considered. Family structure was not explicitly included in the simulation procedure; consequently, the outcomes were based only on the familial relationships present in the parent study. We also did not incorporate the kinship matrix into the GEE and QIF methods as presented. Instead, we made a simplifying assumption of family as the cluster unit and an exchangeable correlation structure. This limitation makes comparisons between LME and GEE/QIF difficult. Important to note, however, is that LME models conducted without the kinship matrix resulted in similar power and Type I error rates as reported above (data not shown). In addition, estimates of heritability are not generated that would have allowed for evaluation of bias. Finally, we did not take full advantage of the correlation structure within subjects and focused instead on a more general multivariate method with repeated measurements. Future research should evaluate longitudinal data with more measurements to determine if these results extend to those settings.

## Conclusions

These limitations outstanding, the present analysis represents one of the first evaluations of QIF models for analyzing genetic data. Our findings suggest that such methods may provide comparable power and adequate control of Type I error rates. Although computational estimates did not include the time necessary for manipulating and cleaning the large data sets incumbent to genetic analysis, these estimates suggest that each model could be scaled up to the epigenomic level. Future studies can further evaluate these models to identify ways to improve computational efficiency for application to large-scale genetic and epigenetic data.

## Abbreviations

BC: Bias-corrected
CpG: Cytosine-phosphate-guanine
GAW: Genetic Analysis Workshop
GEE: Generalized estimating equations
GMM: Generalized method of moments
GOLDN: Genetics of Lipid Lowering Drugs and Diet Network
LME: Linear mixed effects
QIF: Quadratic inference functions
SNP: Single-nucleotide polymorphism
TG: Triglyceride

## References

[1] Liang K-Y, Zeger SL (1986). Longitudinal data analysis using generalized linear models. Biometrika.

[2] Wang Y-G, Carey V (2003). Working correlation structure misspecification, estimation and covariate design: implications for generalised estimating equations performance. Biometrika.

[3] Qu A, Lindsay BG, Li B (2000). Improving generalised estimating equations using quadratic inference functions. Biometrika.

[4] Song PXK (2007). Correlated data analysis: modeling, analytics, and applications.

[5] Song PXK, Jiang Z, Park E, Qu A (2009). Quadratic inference functions in marginal models for longitudinal data. Stat Med.

[6] Westgate PM, Braun TM (2012). The effect of cluster size imbalance and covariates on the estimation performance of quadratic inference functions. Stat Med.

[7] Chen T, Santawisook P, Wu Z (2014). A multi-level model for analyzing whole genome sequencing family data with longitudinal traits. BMC Proc.

[8] Chiu Y-F, Justice AE, Melton PE (2016). Longitudinal analytical approaches to genetic data. BMC Genet.

[9] Chung W, Zou F (2014). Mixed effects models for GAW18 longitudinal blood pressure data. BMC Proc.

[10] Das M, Irvin MR, Sha J, Aslibekyan S, Hidalgo B, Perry RT, Zhi D, Tiwari HK, Absher D, Ordovas JM (2015). Lipid changes due to fenofibrate treatment are not associated with changes in DNA methylation patterns in the GOLDN study. Front Genet.

[11] Kraja AT, An P, Lenzini P, Lin SJ, Williams C, Hicks JE, Daw EW, Province MA. Simulation of a medication and methylation effects on triglycerides in the Genetic Analysis Workshop 20. BMC Proc. 2018;12(Suppl 9). 10.1186/s12919-018-0115-z. 10.1186/s12919-018-0115-z PMC6157129 30275880

[12] Therneau TM, Daniel S, Sinnwell J, Atkinson E. The kinship2 package (for R). R Package. 2015; Available at: https://cran.r-project.org/web/packages/kinship2/kinship2.pdf. Accessed 16 Dec 2016.

[13] Therneau TM. The coxme package (for R): R Package; 2015. Available at: https://cran.r-project.org/web/packages/coxme/coxme.pdf. Accessed 16 Dec 2016.

[14] Westgate PM (2013). A bias correction for covariance estimators to improve inference with generalized estimating equations that use an unstructured correlation matrix. Stat Med.

[15] Mancl LA, DeRouen TA (2001). A covariance estimator for gee with improved small-sample properties. Biometrics.

[16] Hansen LP (1982). Large sample properties of generalized method of moments estimators. Econometrica.

[17] Westgate PM (2012). A bias-corrected covariance estimate for improved inference with quadratic inference functions. Stat Med.

[18] Westgate PM (2014). Criterion for the simultaneous selection of a working correlation structure and either GEE or the QIF approach. Biom J.

[19] Fitzmaurice GM, Laird NM, Ware JH (2012). Applied longitudinal analysis.

[20] Burkett KM, Roy-Gagnon M-H, Lefebvre J-F, Wang C, Fontaine-Bisson B, Dubois L (2015). A comparison of statistical methods for the discovery of genetic risk factors using longitudinal family study designs. Front Immunol.

